# Inferiority feelings mediate the impact of subjective social support on anxiety/depression symptoms in individuals with physical disabilities

**DOI:** 10.3389/fpubh.2024.1417940

**Published:** 2024-11-06

**Authors:** Yiyang Liu, Wenjing Xu, Shanshan Liu, Yuqing Song, Lin Li, Shunfei Li, Hongguang Chen

**Affiliations:** ^1^Peking University Sixth Hospital, Peking University Institute of Mental Health, NHC Key Laboratory of Mental Health (Peking University), National Clinical Research Center for Mental Disorders (Peking University Sixth Hospital), Beijing, China; ^2^Chinese PLA General Hospital, Beijing, China

**Keywords:** disabilities, anxiety, depression, inferiority feelings, public mental health

## Abstract

**Background:**

Persons with physical disabilities are more likely to suffer from psychological symptoms and inferiority feelings, and social support plays an important role in improving those symptoms. However, the interaction between psychological symptoms, inferiority feelings and social support is yet to be understood.

**Methods:**

A cross-sectional study was conducted to investigate anxiety, depression, and inferiority feelings among individuals with physical disabilities in a Chinese sample. The questionnaire included the Generalized Anxiety Disorder 7, Patient Health Questionnaire 9, Self-designed Disability Questionnaire, and Social Support Rating Scale including three dimensions: subjective social support, objective social support and utilization of social support.

**Results:**

Out of the 1,453 respondents with physical disabilities, 49.7, 60.4, and 62.5% reported experiencing anxiety, depression, and inferiority feelings, respectively. Factors such as time since identification of physical disabilities, comorbidities, daily travel, social interaction, internet use, subjective social support, and inferiority feelings were found to be associated with anxiety or depression symptoms among physically disabled individuals. Subjective social support was found to be associated with inferiority feelings, which partly mediated the effect of subjective social support on anxiety symptoms by 37.4% and depression symptoms by 28.7%.

**Conclusion:**

This study highlights the importance of addressing the psychological well-being of physically disabled individuals in addition to their physical rehabilitation. Psychological intervention strategies should focus on improving subjective social support and reducing inferiority feelings, particularly among vulnerable groups.

## 1 Introduction

Disability is a natural part of the human experience, with almost everyone experiencing temporary or permanent disability at some point in their lives (1). According to the WHO Global Report on Health Equity for Persons with Disabilities, released on December 2, 2022, an estimated 1.3 billion people, approximately 16% of the global population, currently experience significant disabilities (2). This number continues to rise, in part due to population aging and the prevalence of non-communicable diseases. In China, around 85 million individuals have a disability or another condition that affects their daily activities and social participation, with approximately 27 million being physically impaired, ranking as the most common type of disability (3). Physical disabilities refer to anatomical and functional impairments of the human motor system that can cause paralysis, limb impairment, or trunk deformity, resulting in varying degrees of activity or participation limitations (4). Furthermore, individuals with physical disabilities are more vulnerable to experiencing psychological issues compared to the general population. Research indicates that people living with physical disabilities are at least three times more likely to experience depression than those without disabilities (5, 6). Moreover, frequent mental distress is associated with poor health behaviors, increased utilization of healthcare services, mental disorders, chronic illnesses, and limitations in daily life (7). When faced with the challenges of physical disabilities, individuals with physical disabilities often experience a range of psychological distress, with anxiety and depression symptoms being more common (8, 9). These psychological issues may not only have a negative impact on their mental health, but also on their social interactions and quality of life. In this context, social support plays a crucial role (10). Social support is not only a source of resources and assistance, but also an emotional support system that can alleviate the psychological pressure faced by individuals with physical disabilities in times of difficulty and challenges, reducing the incidence of anxiety and depression symptoms (11–14). However, in addition to social support, the psychological state of feeling inferior may also play a key role in the mental health issues of individuals with physical disabilities (15). Challenges in self-esteem and self-identity may lead individuals with physical disabilities to develop feelings of inferiority, exacerbating the development of anxiety and depression (16). Therefore, we propose a theoretical framework of mediating effects, exploring the mediating role of feelings of inferiority in the relationship between social support and anxiety/depression symptoms. Based on this theoretical framework, we hypothesize that the impact of social support on anxiety/depression symptoms is not only direct, but may also be achieved through influencing individuals’ feelings of inferiority. Social support: by providing emotional support, social participation, and resources, can improve the mental well-being of individuals with physical disabilities, reducing the extent of their feelings of inferiority. Meanwhile, feelings of inferiority can serve as a psychological mediator, transmitting the effects of social support onto anxiety/depression symptoms. Therefore, through an in-depth exploration of the relationships and mediating mechanisms between social support, feelings of inferiority, and anxiety/depression symptoms, we can better understand the essence of mental health issues in individuals with physical disabilities and provide theoretical support for targeted interventions. This study will contribute to expanding the focus of the mental health field on individuals with physical disabilities, and offer important insights for future research and clinical practice.

## 2 Methods

### 2.1 Study design

The cross-sectional study was conducted between October 1, 2021, and June 30, 2022, in Yuyang District, Shaanxi Province, and Wanli District, Jiangxi Province. With the assistance of the China Disabled Persons’ Federation (CDPF), we contacted individuals with physical disabilities for participation in the study. The CDPF is a national organization that represents people with diverse disabilities, aiming to promote equal participation and inclusion of individuals with disabilities in society. Headquartered in Beijing, the CDPF has approximately 120,000 full-time employees across the country (17). Disability assessment is administered by the CDPF in designated medical institutions, followed by disability registration and the issuance of the Certificate of Disability. To recruit participants for the study, registered physically disabled individuals from both cities were mobilized through QR Codes, which were sent to them via WeChat by the local community disability liaison. Participants can enter the online platform by scanning the QR code to complete this survey.

Subject inclusion criteria are as follows: Aged 18 years and over; Individuals with physical disabilities have been issued a disability certificate; Exclusion criteria are as follows: Individuals with severe visual, auditory, speech, intellectual, or mental disabilities that are unable to cooperate with this survey. Electronic informed written consent was obtained from all respondents before data collection. The study was approved by the Ethics Committee of Peking University Sixth Hospital (Approval number: 2021–32).

### 2.2 Measurements

#### 2.2.1 Sociodemographic data and inferiority feelings

The study utilized a self-designed questionnaire to gather sociodemographic data, including age (<42/≥42&<52/≥52&<60/≥60), gender (male/female), educational level (illiterate/primary or junior/high school/college and higher), marital status (married/unmarried/widowed or divorced), job (yes/no), family’s average monthly income (≤3,000RMB/>3,000&≤5,000RMB/>5,000RMB), number of children (none/one/two and above), religious beliefs (yes/no), cohabitation status (spouse/others/live alone), internet use habits (yes/no), and inferiority feelings (frequency of inferiority feelings due to disability: never, very few, sometimes, always).

#### 2.2.2 Disability-related information

Physical disabilities, per the national standard of classification and grading of disability following the WHO Disability Assessment Schedule 2.0 (WHODAS 2.0), refers to the loss of motor function and limitations in movement or participation caused by structural and functional injuries to the human motor system, trunk paralysis, and deformity. This definition encompasses (1) loss, deformity, or dysfunction of the upper or lower limbs caused by injury, disease, or developmental abnormalities; (2) deformities or dysfunction of the spine caused by injury, disease, or developmental abnormalities; and (3) dysfunction of the trunk or limbs caused by injury, disease, or developmental abnormalities of the central and peripheral nerves. In this study, disability-related information was collected using a self-designed questionnaire. This information included the severity of disability marked on the disability card issued by the local Disabled Persons’ Federation following the national standard of classification and grading of disability, with four levels (I, II, III, IV), of which level I is the most severe. Additionally, the questionnaire asked about the duration since being rated as a physically disabled person, comorbid with other disabilities [In China, disabilities mainly include visual disability, hearing disability, speech disability, physical disability, intellectual disability, and mental disability (18). Here, the term “comorbid with other disabilities” refers to individuals with physical disability alongside at least one of the other five types of disabilities mentioned] and impact on daily travel and social interaction on daily life.

#### 2.2.3 Generalized anxiety disorder 7-item (GAD-7) scale

This scale was used to screen for anxiety symptoms and evaluate the severity, consisting of 7 items. There were four degrees for each item (0-not at all, 1-some of the time, 2-more than half the time, 3-nearly every day). The GAD-7 score ranged from 0 to 21. The higher the total score, the more serious the anxiety symptoms. A total score of 0–4 was rated as no anxiety, 5–9 rated as mild anxiety, 10–14 rated as moderate anxiety, and 15 or more rated as severe anxiety. The scale is widely used in China, with high reliability and validity (19). The Cronbach’s alpha coefficient of the scale in this study is 0.955.

#### 2.2.4 Patient health questionnaire-9 (PHQ-9)

This scale was used to screen for depressive symptoms and evaluate the severity, consisting of 9 items. There were four degrees for each item (0-not at all, 1-some of the time, 2-more than half the time, 3-nearly every day). The PHQ-9 score ranged from 0 to 27. The higher the total score, the more serious the depressive symptoms. A score of 0–4 was rated as no depression, 5–9 was rated as mild depression, 10–14 was rated as moderate depression, 15–19 was rated as moderate to severe depression, and 20 or more was rated as severe depression. The scale is widely used in China, with high reliability and validity (20). The Cronbach’s alpha coefficient of the scale in this study is 0.927.

#### 2.2.5 Social support rating scale (SSRS)

The SSRS was used to assess the current level of overall social support (e.g., from parents, teachers, relatives, and friends) received by the subject. It includes 10 items that measure objective support, subjective support, and support utilization. The questionnaire has been shown to have good validity and reliability in Chinese populations (21). The total SSRS score is the sum of the scores from the three subscales. A higher score indicates receiving more social support. The Cronbach’s alpha coefficient of the scale in this study is 0.846.

### 2.3 Statistical analyses

In this study, several statistical methods were employed to analyze the data. The detection rate was used to describe the proportion of cases with psychological symptoms, and the chi-square test was utilized to compare the distribution of characteristics among different detection rates. To determine the 95% confidence intervals (CI) for the detection rate, the bootstrap method was implemented. Furthermore, we employed ordered logistic regression to analyze the factors associated with psychological symptoms and inferiority feelings. The choice of ordered logistic regression model was based on the fact that our dependent variable is ordinal in nature, such as the levels of psychological symptoms and inferiority feelings. Additionally, social support for psychological symptoms is often influenced, either partially or entirely, by mediators (10). Therefore, this study utilized mediation analysis to explore the potential mediating variables and mediating effect. Mediation effect analyses were performed using a four-step process: (1) examining the association between the independent variable (subjective social support) and the dependent variable (anxiety/depression symptoms); (2) assessing the relationship between the independent variable (subjective social support) and the mediator (inferiority feelings); (3) investigating the association between the mediator (inferiority feelings) and the dependent variable (anxiety/depression symptoms); and (4) establishing the mediating effect by demonstrating that the mediator variable influences the association between the independent and dependent variables. To estimate the indirect effects attributable to inferiority feelings, the STATA binary_mediation command was employed. This indirect effect was calculated as a proportion of the total effect, and the standard errors for the direct and indirect effects, along with a 95% CI, were obtained using 500 bootstrap replications. All statistical tests were two-tailed with a significance level set at *p* < 0.05. Data analysis was performed using STATA 14.0 (Stata Corp, College Station, Texas, United States).

## 3 Results

### 3.1 Sociodemographic and disability characteristics

The study examined 1,491 individuals with physical disabilities. After excluding 38 cases due to incomplete data, the final analysis included 1,453 subjects with a mean age of 50.5 (SD, 13.3) years. Sociodemographic characteristics showed that 63.7% were male, 56.8% had primary or junior high school education, 76.7% were married, 52.3% had urban household registration, and 46.5% had an average monthly household income exceeding 5,000 RMB. Disability information indicated that 46.7% were rated at level IV, 34.7% at level III, 12.7% at level II, and 5.9% at level I, with a median duration of 6.0 years (IQR, 2.9–12.5) since being rated as disabled. Additionally, 24.0% had comorbidities involving other types of disability. The leading causes of physical disabilities were accidental injury (24.6%), osteoarthropathy (14.7%), and spinal cord disease (11.6%).

### 3.2 Prevalence of anxiety, depression, and inferiority feelings

The prevalence of anxiety, depression, and any symptom among individuals with physical disabilities were 49.7, 60.4, and 63.7%, respectively (see Table 1). Most subjects reported mild to moderate symptoms. Notably, significant gender differences were observed, with higher prevalence rates of anxiety and depression symptoms among females (*p* < 0.05). Additionally, 62.5% of respondents reported feelings of inferiority, with 18.2% indicating “seldom,” 29.3% “sometimes,” and 15.1% “always.” Females reported feelings of inferiority more frequently than males (*p* < 0.05). Further analysis revealed that the distribution of characteristics among individuals with anxiety, depression, and inferiority feelings within the disabled population also varies (see Table 2).

**Table 1:** Prevalence of anxiety, depression, and inferiority feelings among 1,453 investigated persons with physical disabilities.

	*N* (%)	Male *N* (%)	Female *N* (%)	*P*
Anxiety				
None	731 (50.3)	495 (53.5)	236 (44.7)	0.004
Mild	361 (24.9)	212 (22.9)	149 (28.2)	
Moderate	184 (12.7)	110 (11.9)	74 (14)	
Severe	177 (12.2)	108 (11.7)	69 (13.1)	
Depression				
None	576 (39.6)	387 (41.8)	189 (35.8)	0.026
Mild	334 (23.0)	203 (21.9)	131 (24.8)	
Moderate	228 (15.7)	152 (16.4)	76 (14.4)	
Severe	171 (11.8)	96 (10.4)	75 (14.2)	
Extremely severe	144 (9.9)	87 (9.4)	57 (10.8)	
Any				
None	528 (36.3%)	359 (38.8)	169 (32.0)	0.010
Anyone	925 (63.7%)	566 (61.2)	359 (68.0)	
Inferiority feelings				
Never	545 (37.5)	374 (40.4)	171 (32.4)	0.002
Very few	264 (18.2)	158 (17.1)	106 (20.1)	
Sometimes	425 (29.3)	274 (29.6)	151 (28.6)	
Always	219 (15.1)	119 (12.9)	100 (18.9)	

**Table 2:** Characteristic distribution of anxiety, depression, and inferiority feelings among 1,453 investigated persons with physical disabilities.

			Anxiety symptom		Depression symptom		Inferiority feelings	
Variable		*N*	Detection rate (%, 95CI)	*P*	Detection rate (%, 95CI)	*P*	Detection rate (%, 95CI)	*P*
Age								
	<42	367	48.5 (43.2–53.9)	0.266	53.4 (47.9–58.8)	0.019	68.4 (63.4–73)	0.000
	≥42 and <52	386	53.9 (49.2–58.5)		62.7 (58.4–66.8)		66.8 (62–71.3)	
	≥52 and <60	358	48.9 (43–54.8)		62.6 (57.9–67)		62.3 (56.4–67.9)	
	≥60	342	47.1 (42–52.2)		62.9 (57.4–68)		51.5 (45.8–57.1)	
Gender								
	Male	925	46.5 (43.3–49.7)	0.001	58.2 (55.2–61.1)	0.024	59.6 (56.5–62.5)	0.002
	Female	528	55.3 (50.8–59.7)		64.2 (59.7–68.4)		67.6 (64–71.1)	
Educational level								
	Illiterate	195	57.9 (50.5–65)	0.007	67.7 (60.2–74.4)	0.000	59 (52.8–64.9)	0.609
	Primary or junior	825	50.2 (46.7–53.6)		63.2 (60.3–65.9)		63.3 (59.6–66.8)	
	High school	233	48.9 (42.3–55.5)		55.4 (48–62.5)		60.9 (53.6–67.8)	
	College and Higher	200	40.5 (33.2–48.2)		47.5 (40.7–54.4)		64.5 (57.8–70.7)	
Marital status								
	Married	1,114	48.5 (45.4–51.6)	0.002	58.8 (56.2–61.3)	0.001	59.7 (57.3–62)	0.000
	Unmarried	162	45.1 (38.7–51.6)		57.4 (51.1–63.5)		69.1 (62–75.5)	
	Widowed or divorced	177	61.6 (54–68.7)		72.9 (65.3–79.3)		74 (67.7–79.5)	
Job								
	Yes	419	41.3 (37.3–45.4)	0.000	52.7 (47.2–58.3)	0.000	57.3 (51.3–63.1)	0.009
	No	1,034	53.1 (49.9–56.2)		63.4 (60.7–66.1)		64.6 (61.6–67.5)	
Family’s average monthly income								
	≤3,000 RMB	581	47 (42.8–51.3)	0.000	54.7 (50.3–59)	0.000	59.7 (56–63.4)	0.003
	>3,000 and ≤5,000	197	38.6 (32.9–44.6)		55.3 (48.1–62.3)		55.3 (49.2–61.3)	
	>5,000	675	55.3 (51.2–59.3)		66.7 (62.4–70.6)		67 (63.8–69.9)	
Number of children								
	None	183	45.4 (39–51.8)	0.444	56.8 (50.1–63.4)	0.400	68.9 (61.1–75.7)	0.057
	One	429	49.9 (45.2–54.6)		59.2 (54.7–63.6)		64.3 (59.4–69)	
	Two and above	841	50.5 (46.8–54.2)		61.7 (58.4–64.9)		60.2 (56.6–63.7)	
Religious belief								
	Yes	96	53.1 (43–63)	0.486	68.8 (57.8–77.9)	0.082	62.5 (52.7–71.4)	0.999
	No	1,357	49.4 (46.8–52.1)		59.8 (57.2–62.3)		62.5 (59.7–65.2)	
Cohabitation status								
	Spouse	1,006	48.4 (44.7–52.1)	0.272	59 (55.9–62.1)	0.206	59.3 (56.3–62.3)	0.001
	Others	296	53.7 (47.5–59.8)		61.8 (55.4–67.9)		70.6 (65–75.6)	
	Live alone	151	50.3 (42.3–58.3)		66.2 (58.4–73.3)		67.5 (59.9–74.4)	
Disability grade								
	Grade IV	679	46.5 (42.2–51)	0.003	58.2 (54.4–61.8)	0.000	58.9 (55.3–62.5)	0.001
	Grade III	504	49 (44.7–53.3)		57.3 (53.2–61.3)		61.9 (57.3–66.3)	
	Grade II	185	56.2 (49.1–63.1)		69.7 (62.6–76.1)		71.4 (63.8–77.8)	
	Grade I	85	64.7 (55.6–72.9)		75.3 (64.6–83.5)		75.3 (62.7–84.7)	
Time duration after physical disabilities								
	<3	543	49.5 (45.8–53.3)	0.167	63 (59.2–66.6)	0.282	63.5 (59.5–67.4)	0.304
	≥3 and <6	190	49.5 (42.3–56.7)		58.9 (52–65.6)		62.6 (55.9–68.9)	
	≥6 and <13	411	46.2 (41.9–50.6)		56.9 (51.5–62.2)		58.9 (54–63.6)	
	≥13	309	54.7 (48.7–60.5)		61.2 (56.1–66)		65.4 (59.4–70.9)	
Comorbid with other disabilities								
	No	1,105	43.8 (40.9–46.8)	0.000	54.9 (52.2–57.6)	0.000	59.5 (56.5–62.5)	0.000
	Yes	348	68.4 (63.4–73)		77.6 (73.2–81.5)		71.8 (66.2–76.9)	
Impact on daily travel								
	No	513	35.5 (31.3–39.8)	0.000	44.4 (40.4–48.5)	0.000	53.2 (49.2–57.2)	0.000
	Yes	940	57.4 (54.6–60.2)		69 (66.1–71.9)		67.6 (64.8–70.2)	
Impact on social interaction								
	No	572	31.8 (27.9–36)	0.000	41.4 (37.7–45.2)	0.000	46.2 (42.2–50.2)	0.000
	Yes	881	61.3 (58.3–64.2)		72.6 (69.6–75.5)		73.1 (70–76)	
Internet use habit								
	No	1,282	50.9 (47.8–54.1)	0.009	59.1 (56.3–61.8)	0.009	63 (60.1–65.9)	0.249
	Yes	171	40.4 (32.8–48.3)		69.6 (63.8–74.8)		58.5 (51.8–64.9)	
Inferiority feelings								
	Never	545	22.2 (19–25.8)	0.000	36 (32.2–39.9)	0.000	–	–
	Seldom	264	46.6 (40.1–53.2)		58 (51.7–64)		–	
	Sometimes	425	67.5 (63.1–71.7)		77.2 (73.4–80.6)		–	
	Always	219	87.2 (82.5–90.8)		91.3 (87.2–94.2)		–	
Anxiety								
	No	731	–	–	27.8 (24.8–31)	0.000	42 (38.8–45.3)	0.000
	Yes	722	-		93.4 (91.6–94.7)		83.2 (80.8–85.4)	
Depression								
	No	576	8.3 (6.5–10.7)	0.000	–	–	39.4 (36.1–42.8)	0.000
	Yes	877	76.9 (74–79.5)		–		77.7 (75.1–80)	
Social support								
	<30	362	65.7 (61–70.2)	0.000	78.7 (73.5–83.1)	0.000	76.8 (72–81)	0.000
	≥30 and <36	306	55.9 (49.8–61.8)		69.3 (63.7–74.3)		74.2 (68.6–79.1)	
	≥36 and <44	420	49.3 (44.3–54.3)		56.9 (51.3–62.4)		60.7 (56.3–65)	
	≥44	365	29 (24.3–34.3)		38.6 (33.7–43.8)		40.5 (34.8–46.6)	

### 3.3 Correlators associated with anxiety, depression, and inferiority feelings

Further multivariable ordered logistic regression analysis results indicated significant associations between comorbidity with other disabilities (see Table 3), limited daily travel, limited social interaction, higher frequency of inferiority feelings and higher levels of anxiety. Internet use habits and subjective social support were associated with a lower level of anxiety. Significant associations were found between comorbidity with other disabilities, limited daily travel, limited social interaction, and a higher frequency of inferiority feelings with depression. Additionally, a longer time since being identified as disabled and higher subjective social support were factors linked to lower levels of depression. Comorbidity with other disabilities and limited social interaction were associated with a higher risk of inferiority feelings. In contrast, higher subjective social support was linked to a lower likelihood of experiencing inferiority feelings.

**Table 3:** Correlators associated with anxiety, depression, and inferiority feelings examined in ordered logistic regression.

Variable	Anxiety symptom*		Depression symptom*		Inferiority feelings^†^	
	Adjusted OR (95%CI)	*P*	Adjusted OR (95%CI)	*P*	Adjusted OR (95%CI)	*P*
Disability grade, grade III	0.96 (0.75–1.22)	0.732	0.88 (0.7–1.11)	0.266	0.97 (0.77–1.21)	0.766
Disability grade, grade II	0.91 (0.65–1.27)	0.564	0.90 (0.65–1.24)	0.523	1.27 (0.92–1.76)	0.150
Disability grade, grade I	1.28 (0.80–2.05)	0.300	1.51 (0.97–2.34)	0.069	1.41 (0.90–2.21)	0.135
Time duration after physical disabilities, year	1.00 (0.99–1.01)	0.754	0.99 (0.98–0.99)	0.027	1.00 (0.99–1.01)	0.779
Comorbid with other disabilities, yes	1.77 (1.37–2.27)	0.000	2.08 (1.62–2.66)	0.000	1.57 (1.23–2.00)	0.000
Influence of daily travel, yes	1.35 (1.03–1.78)	0.032	1.42 (1.10–1.83)	0.007	1.07 (0.83–1.37)	0.619
Influence of social interaction, yes	1.73 (1.32–2.27)	0.000	2.02 (1.57–2.60)	0.000	2.45 (1.91–3.14)	0.000
Internet use habit, yes	0.58 (0.39–0.86)	0.007	1.04 (0.73–1.48)	0.813	0.87 (0.61–1.24)	0.433
Objective social support (scale score)	0.99 (0.94–1.03)	0.546	0.97 (0.93–1.02)	0.214	0.99 (0.95–1.03)	0.699
Subjective social support (scale score)	0.94 (0.92–0.96)	0.000	0.93 (0.91–0.95)	0.000	0.92 (0.90–0.94)	0.000
Utilization of social support (scale score)	1.00 (0.95–1.06)	0.940	0.99 (0.94–1.04)	0.777	0.96 (0.92–1.01)	0.130
Inferiority feelings, sometimes/always	4.88 (3.77–6.32)	0.000	3.79 (3.00–4.79)	0.000	–	–

*Adjusted by age, gender, educational level, marital status, job, family’s average monthly income, number of children, religious belief, cohabitation status and Inferiority feelings. ^†^Adjusted by age, gender, educational level, marital status, job, family’s average monthly income, number of children, religious belief and cohabitation status.

### 3.4 Mediating effect of inferiority feelings on subjective social support and anxiety/depression symptoms

**Figure 1:**
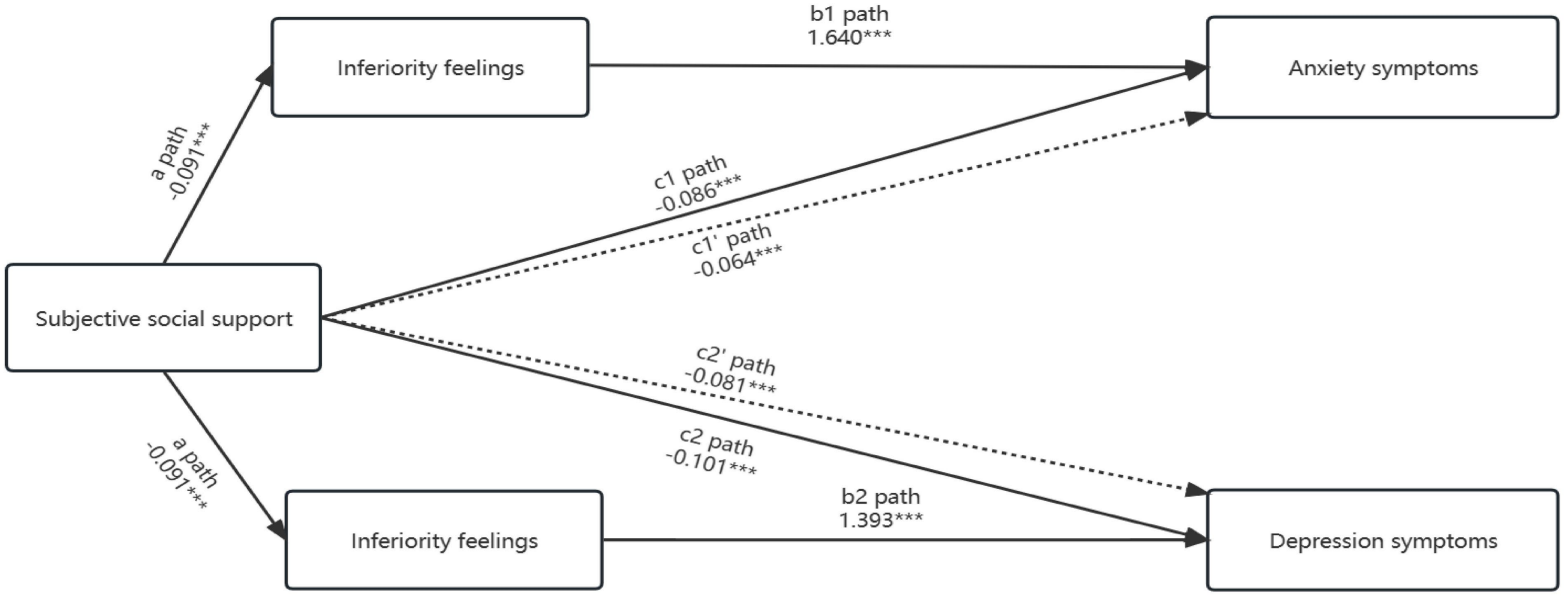
Mediating effect of inferiority feelings on the relationship between subjective social support and anxiety/depression symptoms. Regression coefficients are presented; a path: association between inferiority feelings (mediator variable) and subjective social support (independent variable) examined by logistic regression after adjusting for age, gender, educational level, marital status, job, family’s average monthly income, number of children, religious belief, and cohabitation status, degree of disability grade, time duration after physical disability, other disability comorbidities, daily travel, social interaction, internet use, objective social support and utilization of social support: c1/c2 path: association between anxiety/depression symptoms (dependent variable) and subjective social support (independent variable) examined by logistic regression after adjusting for age, gender, educational level, marital status, job, family’s average monthly income, number of children, religious belief, and cohabitation status, degree of disability grade, time duration after physical disability, other disability comorbidities, daily travel, social interaction, internet use, objective social support and utilization of social support; b1/b2 and c1’/c2’ path: association between anxiety/depression symptoms (dependent variable) and subjective social support (independent variable) and inferiority feelings (mediator variable) examined by logistic regression after adjusting for age, gender, educational level, marital status, job, family’s average monthly income, number of children, religious belief, and cohabitation status, degree of disability grade, time duration after physical disability grade, time duration after physical disability, other disability comorbidities, daily travel, social interaction, internet use, objective social support and utilization of social support; ***p < 0.001.

To further verify the relationship between subjective social support, anxiety/depression symptoms, and inferiority feelings, this study conducted a mediating effect analysis. Figure 1 showed that subjective social support was significantly associated with inferiority feelings (*β* = −0.091, *p* < 0.001), anxiety symptoms (*β* = −0.086, *p* < 0.001), and depression symptoms (*β* = −0.101, *p* < 0.001). Inferiority feelings were independently associated with anxiety symptoms (*β* = 1.640, *p* < 0.001) and depression symptoms (*β* = 1.393, *p* < 0.001). When inferiority feelings were added to the model, the direct effects of subjective social support on anxiety symptoms (*β* = −0.064, *p* < 0.001) and depression symptoms (*β* = −0.081, *p* < 0.001) were both significant, indicating partial mediation. Bias-corrected bootstrap tests with a 95% CI were performed to test the mediating effects (see Table 4). All effects were significant, as the 95% CI did not include zero. The proportion of the total mediated effect was 37.4% for anxiety symptoms and 28.7% for depression symptoms.

**Table 4:** Bias-corrected bootstrap test of mediating effect (*N* = 1,453).

	Anxiety		Depression	
	*β* (se)	95%CI	*β* (se)	95%CI
Indirect effect				
Subjective social support → Inferiority feelings → Anxiety/depression	−0.114 (0.018)	−0.144 to −0.080	−0.098 (0.017)	−0.132 to −0.066
Direct effect				
Subjective social support → Anxiety/depression	−0.191 (0.039)	−0.270 to −0.114	−0.244 (0.040)	−0.328 to −0.171
Total effect	−0.305 (0.039)	−0.374 to −0.205	−0.342 (0.038)	−0.412 to −0.263
The proportion of total effect mediated	37.4%		28.7%	

Bootstrap samples were 500.

## 4 Discussion

This study is among the few that have examined psychological symptoms and feelings of inferiority in individuals with physical disabilities during the COVID-19 epidemic. The prevalence of anxiety or depression was found to be remarkably high at 63.7%, surpassing the rates reported in previous studies conducted by Cree et al. (7) and Jung et al. (22). The increased prevalence can be attributed, at least in part, to the impact of the COVID-19 pandemic. Previous research has demonstrated significant short-term and long-term adverse effects of COVID-19 on the psychological well-being of the general population (23, 24). During the pandemic, individuals with physical disabilities have faced additional challenges such as isolation, disrupted routines, limited access to healthcare services, and decreased social support, all of which have further affected their lives and mental well-being (25). Moreover, variations in measurement approaches, survey locations, socio-demographic characteristics, and levels of disability may contribute to the differences in prevalence rates observed across studies. Consistent with findings from previous (15), a majority of individuals with physical disabilities in this study reported experiencing feelings of inferiority. Inferiority feelings are intricate emotional states that commonly indicate a perception of weakness and helplessness. A lack of timely and effective interventions may significantly affect individuals with such feelings (16). Factors associated with anxiety, depression, and inferiority feelings varied; however, comorbidity with other disabilities, social interaction, and subjective social support were related to all three symptoms. Individuals with multiple disabilities have more limitations in walking, talking, and engaging with peers (26), contributing to their risk of psychological symptoms and inferiority feelings. Those with physical disabilities who have impaired social interaction may have reduced access to social contact and assistance, whereas social engagement has been shown to be effective in improving emotional well-being and reducing the risk of psychological symptoms and disorders. Interestingly, unlike previous studies among young people (27, 28), this study found that internet use was a protective factor against anxiety, which may be because previous studies have primarily focused on problematic internet use or internet overuse among young people. These findings suggest the potential use of online social media as a means to improve the mental health of disabled individuals with limited mobility.

The presence of social support in one’s life has been demonstrated to aid in the development of effective coping strategies, independent living abilities, self-esteem, self-confidence, social interactions, and subjective well-being (29). Social support is a complex construct comprising many facets. This study’s social support rating scale included three dimensions: subjective support, objective support, and support utilization. Unlike previous studies (30, 31), this study found that only subjective social support was significantly associated with a lower risk of psychological symptoms and inferiority feelings. Subjective assessments of received social support are more meaningful than objective measures of social support because they reflect an individual’s psychological perception of reality and can affect their behavior and development. Subjective ratings can capture more subtle aspects of social status not accessible through objective measures, such as income and education, and can better represent factors underlying socioeconomic status differences in behavior and health. Consistent with previous studies (32), this study found that individuals with physical disabilities who frequently or always reported feelings of inferiority had a higher risk of experiencing anxiety and depression symptoms. Due to their sensitivity, people with physical disabilities are more susceptible to external influences and discriminatory perceptions, which can easily cause negative feelings and lower life satisfaction. Previous studies have also shown that more social support can improve self-esteem, reduce perceived discrimination, and improve mental health (15, 33).

Additionally, this study found that inferiority feelings partially mediated the effects of subjective social support on psychological symptoms. The complex interaction between social support and other factors often results in the influence of social support on psychological symptoms and behavior being partly or fully mediated (10). Despite the mediating effect, social support remains an independent protective factor for psychological symptoms, suggesting that psychological support for disabled individuals should be strengthened, especially regarding subjectively perceived psychological support. The results of the mediation analysis further highlight the practical implications for public health. Since inferiority feelings can partially mediate the impact of social support on psychological symptoms, interventions aimed at reducing these feelings should strategically incorporate social support that effectively improves feelings of inferiority. This approach is essential for maximizing the mental health benefits, especially among individuals with physical disabilities. These interventions can substantially alleviate anxiety and depression symptoms by boosting self-esteem and self-worth, facilitating more positive self-images and effective coping strategies. Moreover, acknowledging that subjective social support is inherently personal and perception-based, it is imperative that social support strategies be meticulously tailored to align with the local social and cultural context.

Several limitations of the study should be taken into consideration. First, due to constraints during the COVID-19 pandemic, this study employed a non-probability sampling method instead of a random sampling approach, which may have introduced selection bias. As a result, the findings should be cautiously extrapolated. Second, there is a potential for response bias, particularly social desirability bias, due to the use of self-report scales to measure sensitive topics such as inferiority feelings and psychological symptoms. To mitigate this risk, we implemented anonymous responses. However, despite these precautions, it is important to acknowledge that some degree of response bias may still be present, which could influence the accuracy of the reported findings. Finally, considering the particularity of the survey subjects, the content of the online questionnaire should not be too lengthy. Therefore, a question representing the subjective feelings of the participants was used to reflect the subjects’ feelings of inferiority and the frequency of these feelings. The advantage of this method is that it can quickly obtain an overall self-assessment, while the disadvantage is the inability to assess inferiority in multiple dimensions.

## 5 Conclusion

In conclusion, this study discovered a high prevalence of anxiety symptoms, depression symptoms, and inferiority feelings among individuals with physical disabilities. Several factors were found to be associated with anxiety or depression symptoms, including the duration of time after the onset of physical disabilities, the presence of comorbidities with other disabilities, daily travel, social interaction, internet use, subjective social support, and inferiority feelings. Furthermore, the study revealed that inferiority feelings partially mediated the effect of subjective social support on anxiety/depression symptoms. These findings emphasize the significance of addressing the psychological well-being of individuals with physical disabilities alongside their physical rehabilitation. It is also crucial to develop psychological intervention strategies that specifically target the improvement of individuals’ subjective social support and reduction of inferiority feelings, particularly for vulnerable groups.

## Data Availability

The raw data supporting the conclusions of this article will be made available by the authors, without undue reservation.

## References

[1] World Health Organization. (2023). Disability. Available online at: https://www.who.int/news-room/fact-sheets/detail/disability-and-health#:~:text=Disability%20is%20part%20of%20being%20human%20and%20is,and%20a%20range%20of%20environmental%20and%20personal%20factors (accessed October 15, 2024).

[2] World Health Organization. (2022). Global report on health equity for persons with disabilities: executive summary. Available online at: https://www.who.int/publications/i/item/978924-0063600 (accessed October 15, 2024)

[3] Zheng X Chen G Song X Liu J Yan L Du W. Twenty-year trends in the prevalence of disability in China. Bull World Health Organ. (2011) 89:788–97. doi: 10.2471/BLT.11.089730, PMID: 22084524 PMC3209727

[4] Stucki G Ewert T. Disability and impairment definitions In: Schmidt RF Willis WD, editors. Encyclopedia of pain. Berlin Heidelberg: Springer (2007). 606–11.

[5] Karimi S Andayeshgar B Khatony A. Prevalence of anxiety, depression, and stress in patients with multiple sclerosis in Kermanshah-Iran: a cross-sectional study. BMC Psychiatry. (2020) 20:166. doi: 10.1186/s12888-020-02579-z 32295564 PMC7161227

[6] Noh JW Kwon YD Park J Oh IH Kim J. Relationship between physical disability and depression by gender: a panel regression model. PLoS One. (2016) 11:e0166238. doi: 10.1371/journal.pone.0166238, PMID: 27902709 PMC5130183

[7] Cree RA Okoro CA Zack MM Carbone E. Frequent mental distress among adults, by disability status, disability type, and selected characteristics – United States, 2018. Mmwr Morb Mortal Wkly Rep. (2020) 69:1238–43. doi: 10.15585/mmwr.mm6936a2, PMID: 32914770 PMC7499832

[8] Asdaq SMB Alshehri S Alajlan SA Almutiri AA Alanazi AKR. Depression in persons with disabilities: a scoping review. Front Public Health. (2024) 12:3078. doi: 10.3389/fpubh.2024.1383078, PMID: 38779421 PMC11110534

[9] Steptoe A Di Gessa G. Mental health and social interactions of older people with physical disabilities in England during the COVID-19 pandemic: a longitudinal cohort study. Lancet Public Health. (2021) 6:e365–73. doi: 10.1016/S2468-2667(21)00069-4, PMID: 33894138 PMC8517412

[10] Lin J Su Y Lv X Liu Q Wang G Wei J. Perceived stressfulness mediates the effects of subjective social support and negative coping style on suicide risk in Chinese patients with major depressive disorder. J Affect Disord. (2020) 265:32–8. doi: 10.1016/j.jad.2020.01.026, PMID: 31959583

[11] Akdogan R Cimsir E. Linking inferiority feelings to subjective happiness: self-concealment and loneliness as serial mediators. Personal Individ Differ. (2019) 149:14–20. doi: 10.1016/j.paid.2019.05.028

[12] Gariepy G Honkaniemi H Quesnel-Vallee A. Social support and protection from depression: systematic review of current findings in Western countries. Br J Psychiatry. (2016) 209:284–93. doi: 10.1192/bjp.bp.115.169094, PMID: 27445355

[13] Harvey SB Epstein RM Glozier N Petrie K Strudwick J Gayed A. Mental illness and suicide among physicians. Lancet. (2021) 398:920–30. doi: 10.1016/S0140-6736(21)01596-8, PMID: 34481571 PMC9618683

[14] Newman MG Zainal NH. The value of maintaining social connections for mental health in older people. Lancet Public Health. (2020) 5:E12–3. doi: 10.1016/S2468-2667(19)30253-1, PMID: 31910976 PMC7261393

[15] The Lancet Public Health. Disability-a neglected issue in public health. Lancet Public Health. (2021) 6:e346. doi: 10.1016/S2468-2667(21)00109-2, PMID: 34051158

[16] Liu Y Xu C Kuai X Deng H Wang K Luo Q. Analysis of the causes of inferiority feelings based on social media data with Word2Vec. Sci Rep. (2022) 12:5218. doi: 10.1038/s41598-022-09075-2, PMID: 35338206 PMC8956725

[17] China Disabled Person’s Federation. (n.d.). Introduction. Available at: https://chinadevelopmentbrief.org/ngos/china-disabled-persons-federation/ (accessed October 15, 2024).

[18] Huiyun F Yanan L. Regional patterns of disability and their relationship with socioeconomic conditions — China, 2006. China CDC Wkly. (2022) 4:962–6. doi: 10.46234/ccdcw2022.196 36483791 PMC9713577

[19] Sun J Liang K Chi X Chen S. Psychometric properties of the generalized anxiety disorder Scale-7 item (GAD-7) in a large sample of Chinese adolescents. Healthcare (Basel). (2021) 9:1709. doi: 10.3390/healthcare9121709, PMID: 34946435 PMC8701121

[20] Wang W Bian Q Zhao Y Li X Wang W Du J. Reliability and validity of the Chinese version of the patient health questionnaire (PHQ-9) in the general population. Gen Hosp Psychiatry. (2014) 36:539–44. doi: 10.1016/j.genhosppsych.2014.05.021, PMID: 25023953

[21] Qi RF Luo YF Zhang L Weng YF Surento W Jahanshad N. Social support modulates the association between PTSD diagnosis and medial frontal volume in Chinese adults who lost their only child. Neurobiol Stress. (2020) 13:100227. doi: 10.1016/j.ynstr.2020.100227, PMID: 32490056 PMC7256056

[22] Jung SW Yoon JH Lee W. Predictors for depressive symptoms by four types of disability. Sci Rep. (2021) 11:19371. doi: 10.1038/s41598-021-98765-4, PMID: 34588530 PMC8481329

[23] Chen HG Zhang KL. Insight into the psychological problems on the epidemic of COVID-19 in China by online searching behaviors. J Affect Disord. (2020) 276:1093–4. doi: 10.1016/j.jad.2020.07.128, PMID: 32771861 PMC7395660

[24] Kawakami N Kim Y Saito M Fujishiro S. People's worry about long-term impact of COVID-19 pandemic on mental health. Asian J Psychiatr. (2022) 75:103196. doi: 10.1016/j.ajp.2022.103196, PMID: 35816936 PMC9232257

[25] Shakespeare T Ndagire F Seketi QE. Triple jeopardy: disabled people and the COVID-19 pandemic. Lancet. (2021) 397:1331–3. doi: 10.1016/S0140-6736(21)00625-5, PMID: 33740474 PMC7963443

[26] Xie ZG Tanner R Striley CL Marlow NM. Association of functional disability with mental health services use and perceived unmet needs for mental health care among adults with serious mental illness. J Affect Disord. (2022) 299:449–55. doi: 10.1016/j.jad.2021.12.040, PMID: 34942217

[27] Stiglic G Masterson Creber R Cilar Budler L. Internet use and psychosomatic symptoms among university students: cross-sectional study. Int J Environ Res Public Health. (2022) 19:1774. doi: 10.3390/ijerph19031774, PMID: 35162795 PMC8835365

[28] Xie X Zhu K Xue Q Zhou Y Liu Q Wu H. Problematic internet use was associated with psychological problems among university students during COVID-19 outbreak in China. Front Public Health. (2021) 9:675380. doi: 10.3389/fpubh.2021.675380 34211954 PMC8239128

[29] Cui S Cheng F Zhang L Zhang C Yuan Q Huang C. Self-esteem, social support and coping strategies of left-behind children in rural China, and the intermediary role of subjective support: a cross-sectional survey. BMC Psychiatry. (2021) 21:158. doi: 10.1186/s12888-021-03160-y, PMID: 33731074 PMC7972224

[30] Langlois S Zern A Anderson S Ashekun O Ellis S Graves J. Subjective social status, objective social status, and substance use among individuals with serious mental illnesses. Psychiatry Res. (2020) 293:113352. doi: 10.1016/j.psychres.2020.113352, PMID: 32795772 PMC7669552

[31] Lei X Kantor J. Social support and family functioning in Chinese families of children with autism Spectrum disorder. Int J Environ Res Public Health. (2021) 18:3504. doi: 10.3390/ijerph18073504, PMID: 33800586 PMC8037478

[32] Li JJ Jia SX Wang LS Zhang MM Chen SS. Relationships among inferiority feelings, fear of negative evaluation, and social anxiety in Chinese junior high school students. Front Psychol. (2023) 13:5477. doi: 10.3389/fpsyg.2022.1015477, PMID: 36704691 PMC9872515

[33] Hu FH Zhao DY Fu XL Zhang WQ Tang W Hu SQ. Effects of social support on suicide-related behaviors in patients with severe mental illness: a systematic review and meta-analysis. J Affect Disord. (2023) 328:324–33. doi: 10.1016/j.jad.2023.02.070, PMID: 36813042

